# Effect of mechanical forces on cellular response to radiation

**DOI:** 10.1016/j.radonc.2022.10.006

**Published:** 2022-10-10

**Authors:** Jerome Lacombe, Frederic Zenhausern

**Affiliations:** a Center for Applied NanoBioscience and Medicine, College of Medicine Phoenix, University of Arizona, 475 North 5th Street; b Department of Basic Medical Sciences, College of Medicine Phoenix, University of Arizona, 425 N 5th St, Phoenix; c Department of Biomedical Engineering, College of Engineering, University of Arizona, 1127 E. James E. Rogers Way, Tucson, AZ 85721, USA

**Keywords:** Radiation, Mechanical forces, Stiffness, Shear stress, Mechanotransduction, Mechanoreceptors

## Abstract

While the cellular interactions and biochemical signaling has been investigated for long and showed to play a major role in the cell’s fate, it is now also evident that mechanical forces continuously applied to the cells in their microenvironment are as important for tissue homeostasis. Mechanical cues are emerging as key regulators of cellular drug response and we aimed to demonstrate in this review that such effects should also be considered vital for the cellular response to radiation. In order to explore the mechanobiology of the radiation response, we reviewed the main mechanoreceptors and transducers, including integrin-mediated adhesion, YAP/TAZ pathways, Wnt/β-catenin signaling, ion channels and G protein-coupled receptors and showed their implication in the modulation of cellular radiosensitivity. We then discussed the current studies that investigated a direct effect of mechanical stress, including extracellular matrix stiffness, shear stress and mechanical strain, on radiation response of cancer and normal cells and showed through preliminary results that such stress effectively can alter cell response after irradiation. However, we also highlighted the limitations of these studies and emphasized some of the contradictory data, demonstrating that the effect of mechanical cues could involve complex interactions and potential crosstalk with numerous cellular processes also affected by irradiation. Overall, mechanical forces alter radiation response and although additional studies are required to deeply understand the underlying mechanisms, these effects should not be neglected in radiation research as they could reveal new fundamental knowledge for predicting radiosensitivity or understanding resistance to radiotherapy.

Multicellular life requires a fine regulation to maintain stability of the metazoan tissues and ensure an efficient recovery after the homeostasis dysregulation due to pathological conditions [1]. This regulation has been investigated for decades through the spectrum of the complex interaction between the cells that compose the tissue, including epithelial cells, fibroblasts, endothelial cells and immune cells. As a result, massive data allowed a better understanding of the biochemical signaling that governs this cellular communication.

This intercellular communication has been shown to be a crucial factor of the tissue response to radiation. Although not fully understood yet, the mechanisms behind the bystander effect (the phenomenon wherein non-irradiated cells near irradiated cells also display radiation-induced damages) have been known to involve gap-junction [2] or paracrine communication [3,4]. Another example is the interaction that occurs between the normal cells and the cancer cells within the tumor microenvironment (TME). Thus, the presence of cancer-associated fibroblasts [5,6] or adipocytes [7] have been shown to promote radioresistance of carcinoma cells through secretory mechanisms that regulate DNA damage response.

However, the cells of a tissue or an organ also interact physically with their environment. This mechanical stimulation is sensed by the cells and transformed into a biochemical signal to induce a specific cellular response, in a mechanism commonly referred as mechanotransduction.

Among these physical interactions, the most studied is probably the ubiquitous effect of the non-cellular component of a tissue: the extracellular matrix (ECM). The pioneering work in the early 2000 s, made with the emergence of reliable models [8], highlighted the vital function of ECM stiffness to key cellular processes [9]. Since then, abundance of studies have confirmed this observation, showing that ECM stiffness indeed regulates cellular behavior and metabolism [10] thus affecting tissue functions such as embryonic development and morphogenesis [11,12], homeostasis or disease progression [13–16]. However, many other specific mechanical stimuli may occur in certain tissue, where they are vital for their homeostasis and responsible for their pathological dysregulations. For instance, tensile forces and mechanical strain are important for fetal development, surfactant metabolism, and mechanical ventilation of lung tissue [17], shear flow influences the properties of vascular endothelium [18,19] and plays a key role in activating immune cells and their effector response [20] or long-term compressive forces induced bone mineralization and osteoclastogenesis while oscillation accelerated differentiation of osteoblastic cells [21–23]. The investigation of such mechanical forces is of importance in an ecosystem such as the TME, subjected to several of these stresses that are known to widely alter its phenotype [24,25]. The anarchic cancer cell proliferation within a contained spatial environment generating compressive forces [26], the topological changes of ECM and its stiffening [27–29], the stress generated by the interstitial flow within the tumor niche or produced by the blood flow within the intratumoral blood vessels [30–32], all promote proliferation, invasion and metastasis towards an aggressive phenotype (Fig. 1).

Interestingly, increased lines of evidence also showed that these mechanical cues alter response to chemotherapeutic drug and induce treatment resistance [33,34]. For instance, ECM stiffness has been shown to alter breast cancer cells response to clinically-approved chemotherapeutics as softening the ECM resulted in an increased resistance [35]. By stimulating ERK1/2-dependent expression of RUNX2, compressive stress has also been shown to promote drug resistance in Ewing sarcoma [36]. However, to date, very few studies have investigated the effect of this mechanical environment on the cancer cell response to radiation. Yet, there is a need to understand the role of this biomechanical effect in order to develop new pharmacological approaches to better understand radiation response, enhance radiation treatment (RT), overcome radioresistance or predict radiosensitivity.

In this review, we will present the main mechanotransducers and show their close link with radiation-modulated cellular pathways, thus suggesting that mechanical cues should not be neglected when studying cell response to radiation. We will discuss the few current evidence demonstrating the direct effect of several mechanical stresses on cellular radiosensitivity and discuss the gaps that need to be addressed by the radiation research community in order to improve the current approach.

## Classical mechanotransducers are involved in the modulation of radiation response

### Integrin-mediated adhesion

The interaction between a cell and the ECM occurs through the integrins that are the major metazoan receptors for cell adhesion [37]. These transmembrane proteins make the connection between the ECM proteins and the intracellular cytoskeleton where they can transduce the extracellular signal to activate many signaling pathways. This process includes the phosphorylation of proteins at adhesion sites and the recruitment of various cytoskeletal proteins to form focal adhesions [38]. In total, this dynamic complex involves more than 60 different proteins and is now referred as the cell’s adhesome [39]. Because of this specific configuration, focal adhesions became rapidly a potential candidate as a mechanotransducer that could sense the mechanical forces applied by the ECM onto the cells [38]. Several years of research confirmed this assumption and integrin-mediated adhesion sites are now well recognized as a major player in the sensing of ECM stiffness and elasticity that drive diverse processes such as cell differentiation, proliferation, migration or invasion [40–45].

The interaction between the integrins and the ECM protein network modulates the cell survival after irradiation in a phenomenon called cell adhesion-mediated radioresistance. Integrins are heterodimers formed from 18 α and 8 β transmembrane subunits [37]. Many preclinical studies demonstrated that the β1 integrin, the most common subunit existing in combination among the 24 α/β heterodimers, enhanced cell survival after irradiation and that its inhibition led to an increased radiosensitivity of human head and neck squamous cell carcinoma [46], glioma cells [47], breast tumors [48] leukemia cell lines [49] or normal fibroblasts [50]. When associated with α2 [51] or α5 [52], it demonstrated a preponderant role in promoting invasion of surviving cells. Other heterodimers also showed role in cellular radioresistance, such as αvβ3 integrin in prostate cells [53] or melanoma [54]. Mechanistically, phosphatidylinositol 3-kinase (PI3K)/protein kinase B (Akt) signaling pathway has been identified as the most prominent mediator of the cellular response after α1 integrin ligand binding.

### YAP/TAZ pathway

Recently, a study has identified Yorkie-homologues YAP (Yes-associated protein) and TAZ (transcriptional coactivator with PDZ-binding motif) proteins, the two nuclear effectors of the Hippo signaling pathway, as another major cellular hub of mechanotransduction signaling [55]. In response to ECM stiffness, substrate dimensionality or cell shape, YAP/TAZ is either predominately located in cytoplasm where it is phosphorylated and degraded or translocated into the nucleus where it acts as a transcription factor for a large panel of target genes [56–58]. These target genes encoding for growth factors, cytoskeletal regulators, tissue repair proteins or angiogenic inducers, YAP/TAZ is a master regulator of the cell fate and the maintenance of homeostasis. YAP/TAZ activity is regulated by the conformation and tension of the F-actin cytoskeleton, which is directly impacted by the forces applied by the substrate through the integrins [59]. Therefore, it is important to note the strong interplay between YAP and integrins providing a robust way for cells to differentially respond to dynamic changes [60].

The versatility of YAP/TAZ pathway led to the investigation of its potential role in the radiation response. Overall, preliminary studies showed that YAP conferred radioresistance in many different cells and that tumors with a high YAP expression level had poor prognosis [61–66]. Its inhibition has demonstrated to be a potential therapeutic strategy to enhance RT efficiency since it sensitizes cancer cells to radiation and can reverse aggressive phenotype of radiation-resistant cells that display cancer stem cells properties [67]. Mechanistically, first evidences suggested that YAP was translocated into the nucleus in response to radiation where it promoted FGF2 expression leading to the Mitogen-Activated Protein Kinase (MAPK)–extracellular-signal-regulated kinase (ERK) pathway activation, and ultimately increased cell proliferation while inhibiting apoptosis by accelerating DNA repair and the cell cycle [63]. However, although Fernandez-L *et al*. also showed that YAP activity enhanced cell proliferation, the authors identified that YAP overexpression in medulloblastoma cells prevented complete DNA repair and promote inactivation of the checkpoint regulators ATM and Chk2 leading to the blockade of the activation of the G2/M checkpoint and to a mitosis entry with un-repaired DNA [61]. They suggested insulin-like growth factor 2 (IGF2) expression and Akt activation as a YAP target for the initiation of the radiation response. Another study highlighted that only TAZ level is reduced after irradiation while the expression level of its paralog YAP stayed unchanged [68]. Interestingly, the authors demonstrated that this TAZ inhibition promoted radiation-induced senescence and is triggered by the β-catenin destruction complex in the Wnt pathway, independently of the Hippo pathway. At the sight of these contradictory data, it is evident that the mechanisms of action of YAP upon irradiation need further investigation, but it also revealed the significant role of this pathway in the radiation-induced cell death.

### Wnt/β-catenin signaling

The β-catenin–dependent mechanotransduction is a 600 million year regulatory mechanism, identified in many animal mesoderm [69,70]. Several lines of evidence highlighted the role of strain [71], shear stress [72,73], and mechanical loading [74] on the activation of the Wnt/β-catenin pathway. This response involves both the canonical and non-canonical pathways as well as the cadherin-mediated signaling [75]. Although the role of ECM stiffness is not well understood [76], preliminary work suggests that an increased stiffness promoted the expression of several members of the Wnt/β-catenin pathway [77]. Interestingly, authors showed that the activation of β-catenin was dependent on the activation of integrin/FAK pathway but not on Wnt signals and was involved in the differentiation of mesenchymal stem cell and the maintenance of primary chondrocyte phenotype.

Ionizing radiation promotes accumulation and translocation of β-catenin into the nucleus, leading to an increased transcriptional activity of β-catenin target genes and up-regulation of proteins in the Wnt/β-catenin pathways [78]. The activation of these downstream pathways has been shown to enhance invasion, migration, proliferation, DNA damage repair and thus induce radioresistance [79]. This is all the more important for progenitor/stem cells where Wnt/β-catenin signaling is activated after irradiation, impacting their self-renewal and thus promoting radioresistance to cancer treatment and recurrence [80,81]. Consequently, inhibition of Wnt/β-catenin has been explored to enhance RT and increase tumor radiosensitivity [82]. The underlying mechanisms of this radiation-induced Wnt/β-catenin-dependent cellular response still remain unclear and only few studies have identified either upstream regulators such as glycogen synthase kinase 3β (GSK3β) [83] or downstream effectors such as gelatinases (matrix metalloproteinases (MMP) 2 and 9) [78] as responsible for the radioprotective effect.

### Other mechanoreceptors

#### Mechanosensitive ion channels

The ion channels present on the cell membrane can sense the external mechanical forces applied to it and thus play a major role in the mechanotransduction [84]. Among the high diversity of ion channel types, some of them have been shown to be mechanosensitive such as the TREK and TRAAK subfamily of two-pore-domain potassium (K2P) channels [85], transient receptor potential (TRP) channels [86] or sodium/amiloride-sensitive degenerin (ENaC/DEG) family [87]. More recently, the non-selective calcium-permeable channels PIEZO1 and PIEZO2 have also been identified as mechanoreceptors in response to shear stress, membrane tension, and pressure [88,89]. Because they mediate the transport of inorganic ions like Na^+^, K^+^, Ca^2+^, or Cl^−^ between the extra- and intracellular medium, these channels are vital for cell interaction with the environment and are implicated in many cellular functions such as cell cycle regulation, apoptosis, oxidative defense, metabolism, etc., thus ensuring cellular homeostasis. Ionizing radiation being known to modulate their activity, the role of ion channels in the regulation of cellular radiosensitivity has already been investigated and extensively reviewed by Huber *et al* [90,91].

#### G protein-coupled receptors

The G-protein-coupled receptors (GPCRs) superfamily is the largest group of membrane receptors in humans. The binding of a ligand to the extracellular domain of the GPCR activates downstream effectors and stimulates signaling pathways inside the cell to trigger a large variety of processes involved in the regulation of the cellular phenotype and response to external stimuli. Early studies reported that mechanical stress promoted the release of small molecules that would bind and activate the GPCRs such as the stretch-induced autocrine secretion of angiotensin II (Ang II) in cardiac myocytes that binds Ang II receptors to act as an initial mediator of the cellular response [92]. However, additional evidence showed that the GPCRs mechano-response could also be independent from ligand binding and that mechanical forces could directly activate GPCRs by inducing conformational changes. Thus, Zou *et al*. demonstrated that mechanical stress activated Ang II receptors without the involvement of Ang II but by the association with Janus kinase 2 and translocation of G proteins into the cytosol [93]. New evidence showed that shear stress induced conformational transitions of GPCRs thus revealing their ability to directly sense mechanical forces [94,95]. Recent studies demonstrated that a subfamily of GPCRs, called adhesion GPCRs, actually detect mechanosensory information through their extracellular domain attached to the extracellular matrix [96,97]. Interestingly, under mechanical force, adhesion GPCRs may influence the activity of other transmembrane proteins, such as TRP channels, thus demonstrating that a same mechanical signal could affect different mechanoreceptors that can then interact and induce a synergetic cellular response to this stress [98,99]. Despite the broad range of external signal sensed by GPCRs, their potential role in the radiation response has not been extensively investigated and remains uncertain. Nevertheless, ionizing radiation has been shown to increase expression level of GPCR-encoding genes [100,101] suggesting that cellular response to radiation could be altered by GPCR signaling pathways. However, to date there is few direct evidences of such implication. Franco *et al*. demonstrated that the overexpression of G protein-coupled receptor kinase 2 (GRK2) protected human embryonic kidney cell line against radiation [102]. Specifically, subcellular localization of GRK2 is modified by radiation with GRK2 locating at the membrane shortly after irradiation (3 h) while accumulating preferentially in the mitochondria at longer time points (8 h post-irradiation) resulting in alteration of mitochondrial morphology, mass and function. Overexpression of GRK2 restores full mitochondrial recovery by binding key molecules involved in the process of mitochondrial fusion and recovery: MFN-1 and 2. An indirect evidence of GPCR involvement in cellular radiation response is also the role of the G proteins in the induction of apoptosis after 10 Gy gamma ray-irradiation by up-regulating Bak expression via CREB and AP-1 [103].

## Mechanical stress affects cellular radiosensitivity, but mechanisms are unclear

### ECM stiffness

The close correlation between the activation of mechanotransducers and the modulation of cellular radiosensitivity described previously could reveal a potential and significant effect of mechanical stress on radiation-induced tissue damage and response. However, to date, few studies have really investigated the direct effect of mechanical cues on cellular response to radiation. Two recent studies have focused on the effect of ECM stiffness on the *in vitro* radiation response of cell lines [104,105] (Table 1). In both studies, cells seeded on low stiffness hydrogels (0.5–1 kPa) showed decreased survival and increased apoptosis after irradiation compared to higher substrate stiffness (>5 kPa). Deng *et al.* investigated deeper the mechanisms involved in this response and observed that both homologous recombination (HR) and non-homologous end joining (NHEJ) pathways were inhibited in low stiffness environment, suggesting that double-strand breaks (DSBs) repair can indeed be regulated by extracellular mechanical signals [105]. They further reported that this DSB repair inhibition was under the influence of Rap2-MAP4K4/6/7-ubiquitin signaling. Authors suggested that in low stiffness condition Rap2 activated MAP4K4/6/7 kinases leading to the phosphorylation of ubiquitin and thus preventing the initiation of RNF8-mediated ubiquitin signaling and the recruitment of DSB repair effectors such as RNF168, 53BP1 and BRCA1. At this stage, it is worth noting that MAP4K4/6/7 kinases regulate downstream signaling LATS-YAP pathway, in parallel of MST1/2, by phosphorylating and activating LATS1/2 kinases leading to the subsequent phosphorylation of YAP/TAZ [106]. However, authors also showed that the depletion of LATS1/2 or YAP/TAZ affected cellular radiation response in a similar way as control cells, suggesting that the inhibition of DNA repair by MAP4K4/6/7 kinases at low stiffness is independent of LATS-YAP pathway.

In our opinion, these results should be placed in parallel to the increasing evidence showing the relationship between environmental stiffness, chromatin state and cellular phenotype. Chromatin structure is an important regulator of gene expression and thus dictates many biological processes such as stem cell differentiation [107]. Chromatin accessibility is regulated by chemical interaction such as enzymatic modifications (e.g., acetylation, methylation, etc.) of specific histone proteins [108]. However, the discovery that ECM stiffness influenced lamin-A protein levels [109] highlighted the mechanoresponse of the nuclear lamina and served as a fundamental base to establish a link between mechanical cues and chromatin remodeling. Additional studies have since brought direct evidence of such mechanisms. Using photosoftening hydrogels, Killaars *et al*. thus demonstrated that cells in soft environment displayed a lower nuclear volume, a higher nuclear sphericity, more condensed chromatin and a decreased histone deacetylase (HDAC) level compared to stiffer substrate [110]. Interestingly, they also showed that this chromatin reorganization adapted rapidly after softening and can be reversible or irreversible depending on time of exposure to stiff microenvironments. Stowers *et al*. highlighted that stiff ECM induced a tumorigenic phenotype of breast spheroids [111]. These changes are dictated by the modification of chromatin state, cells in stiff environment having more accessible chromatin sites, increased lamina-associated chromatin and cells in soft condition producing more physiologically representative chromatin accessibility profiles. They demonstrated that this stiffness-induced tumorigenic phenotype is under the control of Sp1-HDAC3/8 mediated pathways. In line with these observations, it is important to mention the clear association between chromatin accessibility and damage response, and in particular radiation-induced damages. Indeed, compacted chromatin has been shown to be less susceptible to radiation-induced DSBs and DNA damages than decondensed domains [112–116]. A recent study confirmed these results *in vivo* by showing that the irradiation of cells with a low number of nucleosomes produced more DNA breaks [117]. The analysis of nucleosome positioning and occupancy with the location of DSBs revealed that nucleosome shielded DNA with DSBs occurring mainly in nucleosome-free regions. Without direct link with ECM stiffness, another example of the effect of chromatin structure on the formation of radiation-induced DNA damages is the three-dimensional (3D) culture of cancer cells that showed increased levels of heterochromatin, characterized especially by histone H3 deacetylation, resulting in increased survival and decreased DSBs numbers and lethal chromosome aberrations [118]. Altogether, these data led to the emergence of a new strategy to enhance tumor radiosensitivity by inhibiting HDACs [119]. The decrease of HDACs activity enhances histone acetylation and promotes formation of uncondensed chromatin more prone to radiation-induced damages.

The observation of a soft environment promoting the formation of a condensed chromatin less susceptible to radiation-induced damages suggests that cells on soft substrate should be more radioresistant and thus contradicts the preliminary results previously described and obtained with hydrogels of different stiffness. Additional investigations are thus required to draw a complete overview of the multiple mechanoresponsive targets, identify their interactions and their respective influence in the cellular response to eventually understand the underlying mechanisms that affect the stiffness-mediated radiosensitivity. Mechanosensing has been mainly studied using two-dimensional (2D) models, and it is now essential to move into a more physiologically relevant 3D context as 3D cellular structures exhibit major mechanoresponsive features such as increased integrins expression and ECM interaction.

Beyond radiosensitivity, Panzetta *et al*. also investigated the adhesion and migration response to radiation of breast normal (MCF10A) and cancer (MDA-MB-231) cells seeded on physiological (1.3 kPa) and malignant (13 kPa) matrix stiffness [120] (Table 1). Results showed that stiffer environment increased cellular and nuclei area of MCF10A but not MDA-MB-231. After radiation, normal cell spreading decreased in a more significant way on stiff substrate while cell spreading of metastatic cells increased on stiff matrix 3 days after 10 Gy. Authors also showed that cell motility in response to radiation is affected by the environment stiffness. Migration velocity of MCF10A cells decreased with the stiffness and conversely, MDA-MB-231 motility and directional persistence increased with the increase of stiffness. Irradiation had a strong impact on MCF10A motility, especially for cells on stiff substrate whose migration velocity and directional persistence increased. MDA-MB-231 on stiff substrate had a similar response to radiation but cells on softer matrix displayed decreased motility and increased directional persistence. Overall, although they did not assess radiosensitivity by clonogenic or proliferation assays, the authors concluded that softer matrix stiffness may protect cells from radiation by preventing cell motility and invasion. The differences observed between normal and malignant cells also suggested that the effect of stiffness on radiation response may also be dependent on the cell metabolism.

### Shear stress

Shear stress is the force that is applied parallel to a material cross-section. In the body, shear stress is mainly prominent in the blood vessels where the mechanical frictional forces created by the blood flow create a stress along the blood vessel wall. As a natural component of the blood vessel physiology, this flow-induced stress is known to have an important regulatory effect on the phenotype of the endothelial cells [19]. Indeed, shear stress has been shown to regulate permeability and barrier function [121–123], cell migration through integrin signaling [124], alignment and polarity [125–127], thus affecting functions such as short-term vasoreactivity, long-term vessel remodeling, and vessel architecture building during vascular morphogenesis [128].

However, although less important in magnitude, epithelial cells can also be exposed to shear stress forces when fluid is present. For instance, in the kidney, the flow of glomerular filtrate creates a significant fluid shear stress at the apical surface of the renal tubular epithelial cells [129]. This stress has been shown to alter epithelial organization and barrier function by modulating expression of cell junction proteins [130], protein uptake by regulating apical endocytosis [131] or cell volume by inducing autophagy [132]. Similarly, the recent use of organ-on-chip demonstrated the effect of physiological shear stress on intestinal cells resulting from the peristalsis. Such constraint has shown to induce a high resistance of the intestinal barrier [133], promote villus differentiation [134], and increase several biochemical activities such as mucus production, vacuolization, mitochondrial metabolism, etc. [135]. Interstitial cells are also under the effect of the interstitial fluid movement. Although very slow (i.e., 0.1–4.0 μm s^−1^), this flow generates a significant shear stress (comprised between 0.001–0.01 dyne/cm^2^) due to the high resistance of the surrounding 3D ECM. This fluidic stress has important roles on cell–cell and cell-ECM signaling driving tissue development and maintenance or pathobiology [136,137]. In cancer biology, the ascitic fluid flow in the peritoneal cavity, triggered by gravity, bodily movements, change in the diaphragmatic pressure from breathing, and organ movements [138], also affects the behavior of ovarian cancer cells. The exposure of tumorigenic cells to shear stress indeed enhanced anchorage-independent survival, induced cytoskeleton changes and chromosomal instability, suggesting a role in promoting invasion and metastasis [139,140].

The mechanotransduction of shear stress shares similarities with the one involved in ECM stiffness response, and several common mechanosensors are triggered in both stresses. Indeed, integrins for example are also activated by shear stress that switches them to an active conformation thus increasing their affinity for ECM proteins [141]. This activation then initiates the phosphorylation of effector proteins through FAK/c-Src and Cav-1/Fyn pathways that leads to a Raf-MEK-ERK response. Therefore, it was foreseeable that shear stress could regulate radiosensitivity by modulating integrins/FAK signal pathway. Luo *et al*. showed that a prolonged (24 h) shear stress of 12 dyne/cm^2^ enhanced the radiation induced cytotoxicity to colon cancer cells [142] (Table 1). The mechanistic study that the authors conducted revealed that combined shear stress and radiation down-regulated FAK protein through the cleavage of caspase 3 and 8 followed by ubiquitin-dependent proteosomal degradation. Consequently, both Akt and cortactin signal were suppressed but no significant change in Erk, ILK, and GSK3β pathways were observed. These results highlighted a certain effect of high shear stress on cancer cells. However, it is worth noting that a shear stress of 12 dyne/cm^2^ is elevated and it is very unlikely that tumor cells are exposed to such high flow stress *in vivo*. The authors showed that lower shear stress (<3 dyne/cm^2^) did not affect cell survival after radiation, therefore, it is still to be determined if physiological shear stress can really affect cancer cells radiosensitivity.

However, high shear stress is encountered in vascular system where it can be comprised between 1 to 6 dynes/cm^2^ for veins to up to 70 dynes/cm^2^ for arteries and capillaries [143]. Thus, Natarajan *et al.* investigated how the hemodynamic flow shear could influence the radiation response of the vascular endothelium [144] (Table 1). Authors showed that in human aortic endothelial cells, the presence of a 16 dyne/cm^2^ shear stress induced a higher constitutive level of endothelial nitric oxide synthase, decreased radiation-induced oxidative stress, induced transient NF-κB activation (while low shear stress (2 dyne/cm^2^) triggered a sustained NF-κB activity), and activated stem cell biology signaling pathways. The extensive investigation of shear stress effect on endothelial cells over the years also highlighted specific mechanoreceptors such as tyrosine kinase receptors (TKRs), G proteins and GPCRs, ion channels, intercellular junction proteins (e.g., VE-cadherin and PECAM-1), caveolae, membrane lipids, and glycocalyx [18,145]. Therefore, there is a need to define the role of these additional mechanotransducers that could contribute to affect radiation response of endothelial cells and thus help to prevent radiation-induced cardiovascular injuries.

Following the discovery of its role in ECM sensing, the role of YAP in shear stress transduction signaling is also now under investigation. Preliminary data showed that fluid shear stress activates ROCK and LIMK. Once activated, LIMK phosphorylates cofilin that inhibits actin depolymerization leading stabilized F-actin filaments. In such circumstances, YAP is dephosphorylated, moves to the nucleus where it interacts with TEAD and acts as a transcription factor for genes promoting motility and survival in cancer cells [146]. To note that TAZ protein level was also slightly elevated under shear stress stimulation, but its inhibition did not induce cell movement, suggesting that despite its shear stress-induced activation, TAZ has a distinct function in mechanotransduction than YAP. In the meantime, using zebrafish model, another study demonstrated that *in vivo* blood flow induced-shear stress enhanced nuclear import of YAP through regulation of F-actin and AMOT thus participating in the maintenance of blood vessels [147]. Since then, many evidence confirmed that YAP activation by shear stress induced cellular phenotypic changes including breast cancer cell proliferation via ERK phosphorylation [148] or epithelial-mesenchymal transition in hepatocellular carcinoma [149]. Importantly, a study demonstrated that the nature of the flow could guide YAP/TAZ response [150]. Wang *et al*. thus showed that disturbed flow (without a clear direction) only, and not laminar flow (with a clear direction), led to YAP/TAZ activation in endothelium, suggesting that YAP activation by fluid stress is a fine and complex regulated mechanism and highlighting the need of a better understanding of the role of the mechanoreceptors involved in this mechanotransduction response.

### Mechanical stretching

Several tissues in the body are subjected to mechanical stretch including lung cells in the alveoli during breathing movement, cardiac tissue during heart beating, blood vessels due to blood flow and the action of blood pressure, muscle during body movement, intestinal cells during intestinal peristalsis or bladder at the time of urination [151]. With the improvement on the design of cell stretching devices, the study of such mechanical stress has expanded these last years along with our knowledge [152]. Through the stretch-activated cation channels, it is now well-established that mechanical strain induces an intracellular calcium influx that triggers a signaling mechanotransduction cascade controlling a large array of cellular functions [153,154]. The stretching-induced cellular response also involves major mechanotransducers previously described such as integrins [155] or YAP/TAZ pathway [156]. Interestingly, a recent study demonstrated that integrin-based adhesions deformation under stretching was not due to the direct action of external force but by the transient and local displacement of the cytoskeleton [157].

To date, we have not identified any studies that investigated the role of mechanical strain on cellular response to ionizing radiation. However, using mouse fibroblasts seeded on an elastic polydimethylsiloxane (PDMS) membrane, Nagayama and Fukuei showed that 10 % cyclic uniaxial stretch at a frequency of 0.5 Hz for 12 h decreased γH2AX fluorescence compared to non-stretched control after ultraviolet (UV)-C irradiation at 45 J/m^2^ [158] (Table 1). Interestingly, this response seemed to be dependent of the strain amplitude as a 4 % cyclic stretch displayed a significantly higher γH2AX level than the 10 % stretch. However, authors did not investigate further the mechanisms that could explain such observation and did not assess if stretching modify cellular sensitivity to UV. Although UV is not considered ionizing radiation, the difference in γH2AX level, a biomarker of double-strand breaks DNA, may suggest that mechanical strain could indeed affect radiation-induced DNA damage response, and so radiosensitivity.

Mechanical stretching of cells is sensed by the cells through the membrane receptors and can be transduced to the nucleus through the remodeling of the cytoskeleton linking the plasma and nucleus membrane [159]. This cell external stretching force sensed by the nucleus induces then chromatin stretching and conformation changes leading to transcription upregulation [160,161]. As previously described for the stiffness sensing, since mechanical strain promotes chromatin compaction, it is reasonable to think that such stress could thus decreased radiation-induced DNA damages level and protect cells from radiation.

## Ionizing radiation also affects tissue mechanical properties

Although this review aims to discuss the effect of mechanical properties on the cellular radiation response, it must be noted that radiation can also induce mechanical changes in the targeted tissue. When used at high dose for sterilization purpose of decellularized tissue, irradiation has been shown to drastically decrease Young’s modulus, yield strength and ultimate tensile strength of rabbit kidney [162]. In porcine kidney, such doses also lowered both collagen and sulfated glycosaminoglycans content and damaged collagen fibers increasing void space in the matrix [163]. However, contradictory data have been gathered from porcine decellularized cornea, where a 25 kGy γ-irradiation appeared to increase tensile strength, elastic modulus and compressive modulus compared to non-irradiated decellularized tissue [164]. For more relevant clinical radiation doses (10–100 Gy), it has been shown that such doses decreased tissue stiffness [165] and increased the elastic modulus [166] of tumors. To explain such observations, investigations focused on the radiation-induced structural changes and composition alterations that may occur in the main ECM proteins [167,168]. Thus, the stiffness and tensile modulus of collagen decreased after irradiation, and although radiation seems to not affect matrix architecture and to not denature collagen, it has been hypothesized that radiation-induced cleavage of the C–C and C–N bonds could destabilize the collagen triple helix and explain the alteration of ECM mechanical properties [165]. For elastin, the elastic modulus decreased significantly after 10 Gy-irradiation (and up to 50 Gy), suggesting the probable occurrence of chain breakages [166]. Radiation also increased production of hyaluronic acid (HA), another key component of the ECM, leading to changes in ECM biochemical tension [169], and decreased its viscosity, thus potentially impairing its function as lubricant [166]. However, these changes are likely to be tissue specific. For instance, a study also observed an increased stiffness in radiation-treated skin tissue, but, although showing an increase in tenascin expression, did not highlight changes in elastic fibers/collagen [170]. Another example showed that for bone tissue, only high, non-physiological relevant doses (17–35 kGy) were found to decrease monotonic strength and to induce collagen fragmentation, compared to non-irradiated or low dose-irradiated samples [171].

Despite some conflicting data potentially due to the nature of the targeted tissue, the different radiation protocols or the variety of mechanical testing approaches, there is clear evidence that radiation also affects the mechanical properties of the tissue. This highlights a complex reciprocal interaction where radiation can modify mechanical cues but where these same mechanical forces also affect the tissue radiation response. In addition, beyond the mechanical alterations, radiation can also directly affect the chemical components of the cell and so potentially the structure and composition of the mechanoreceptors used to sense the radiation-induced mechanical modifications. The need of better understanding these complex interactions, and determining if the effect of radiation on ECM is synergetic, complementary or exclusive to the effect of ECM on radiation response, is vital, as it could potentially explain certain relapse or bystander effects and ultimately drive clinical management. For example, if radiation indeed stiffens ECM and that high stiffness drives cells towards a radioresistant phenotype, alternative therapeutic should be explored depending on TME characteristics.

## Conclusion and perspectives

**Fig. 2.**
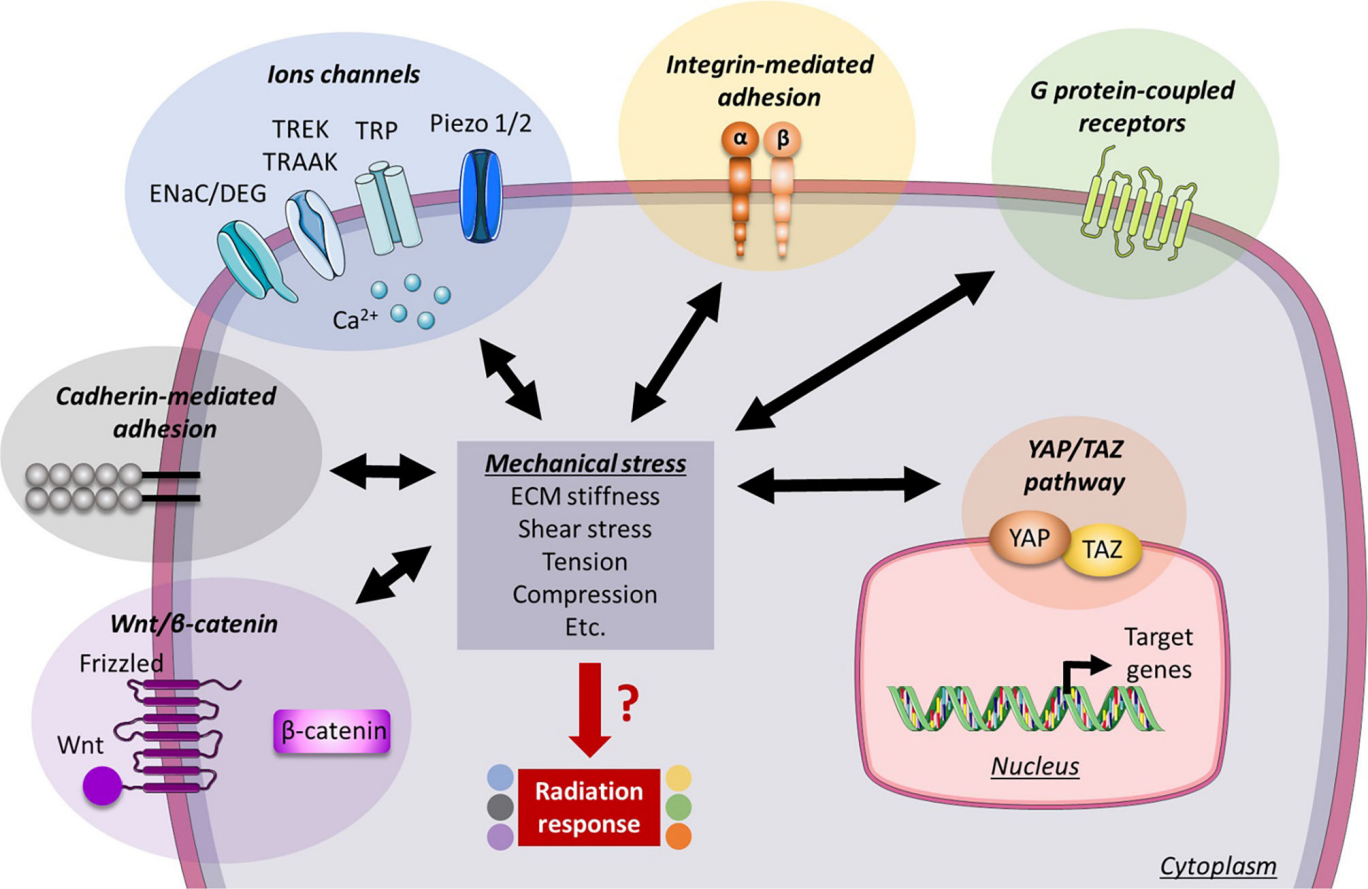
Mechanical forces such as stiffness, shear stress or tensile and compressive forces are sensed by cellular mechanoreceptors that can transduce mechanical signal into biochemical response. The six main families of the mechanotransducers used by the cell to sense these forces are also known for their role in the modulation of radiation response, through diverse mechanisms such as cell adhesion-mediated radioresistance or ligand-mediated mechanotransduction. An emerging question is to understand if the mechanical forces can thus directly affect cellular radiosensitivity.

It appears clearly that the main receptors sensing external mechanical forces and the cellular pathways transducing this signal are also involved in the modulation of the cellular radiation response (Fig. 2). Despite a lack of reliable direct evidence, it is therefore conspicuous to assume that mechanical forces can play an important role in the radiosensitivity status of a tissue. The few studies that already investigated cell’s fate after irradiation as a direct consequence of changes triggered by mechanical stresses showed that mechanical cues indeed modify cellular radiosensitivity. Either if the increase in mechanical load radio-protect or sensitize tissue, or by which mechanisms these mechanical forces modify the radiation response, the answers to these questions are still unclear as the preliminary results appear paradoxical. This contradiction is probably the consequence of the complex environment in which the cells develop and the multiple interactions that both irradiation and mechanical stress can trigger within the tissue. For example, the attempt of combining RT with integrin inhibitor monotargeting in invading tumors led to a high rate of relapse and metastasis, and a recent study highlighted a better efficacy when targeting several integrin subunits [172], suggesting that a potential crosstalk and feedback regulation altering the biological response could occur between the different mechanoreceptors in response to either radiation and/or environment. To investigate such effect, there is a need for more developed models that should consider both chemical and mechanical cues.

Indeed, the effect of mechanical cues on cellular sensitivity to therapeutic drug revealed that standard research models using unnaturally rigid substrate can lead to unreliable *in vitro* drug responses [35]. Regarding the clear impact of these mechanical forces on radiation response, it is important to highlight that radiation research also needs to consider implementing more mechanically relevant models. For instance, using decellularized vegetal scaffolds with mechanical properties (i.e., stiffness, elasticity, topography, etc.) similar to human tissue [173], our lab showed that cells seeded on such matrix displayed a different gene profile for DNA damage signaling pathways compared to standard cell culture models after irradiation, highlighting the importance of mechanical factors when assessing radiation response and the critical choice of the biological model [174]. The use of bioengineered approaches and microfluidics technology would also benefit the field and already demonstrated their usefulness [175]. For example, in the past decade, organ-on-chip technology has emerged as a reliable approach to mimic complex microenvironment by integrating multiple cell types in a mechanically regulated environment [176]. Using the deformability property of PDMS to make a lung- [177] and glomerulus-on-chip [178], Ingber’s group showed the importance of mechanical strain, mimicking respectively breathe movement and cyclic pulsations of renal blood flow, in the biological function of the tissue and emphasized the importance of incorporating these effects when studying drug response [179]. The integration of mechanical stimuli in organ-on-chip is a growing field [180,181] making this technology a robust *in vitro* model to reproduce the complex biochemical and mechanical of the human *in vivo* environment and study complex response to external stimuli such as irradiation.

Finally, identifying the role of mechanical cues in radiation response could lead to new complementary medicines for RT. To date, there already are anti-cancer therapeutic approaches targeting mechanical properties of ECM. These approaches consist on softening the ECM or decreasing the cellular interaction with the stiff ECM either by degrading or inhibiting the synthesis of its main components (i.e. collagen or HA) or by targeting the cell-matrix junctions and the mechanotransduction response (i.e., integrins, FAK, Rho signaling, etc.), respectively [13,182]. Whether future researches reach a consensus stating that soft ECM indeed radiosensitizes tumor, such complementary approach could also then become of great value for RT. Even further, drug systems can now be designed and tuned to be mechanically activated. These mechanical force-responsive drug delivery systems use compression, strain or shear stress as stimulus triggers to allow a controllable release of the bioactive compounds at a specific tissue area [183,184]. An advanced personalized medicine could use such systems to release radio-sensitizers, protectors or mitigators in the targeted tissue that display unique mechanical features. For instance, the physical forces generated during the tumor development compress blood and lymphatic vessels, leading to a reduction of perfusion rate that generates hypoxia and ultimately promote radioresistance. The narrowing of these vessels causes significant changes in shear stress that could be used to specifically unload a radiosensitizer drug to the tumor through a shear-stress sensitive drug delivery system.

The permanent discovery of new molecular partners as receptors or transducers of the mechanical response, conjugated with the continuous improvement of bioengineered models should encourage the radiation research community to investigate the role of mechanical cues as such effects might clearly influence tissue radiosensitivity, resistance to treatment and bystander effects, among others radiation-responsive cellular processes. Ultimately, a comprehensive knowledge of these mechanical-modified radiobiological phenomena will allow the emergence of new treatments and alternative strategies in RT that could greatly benefit the patients.

## Figures and Tables

**Fig. 1.**
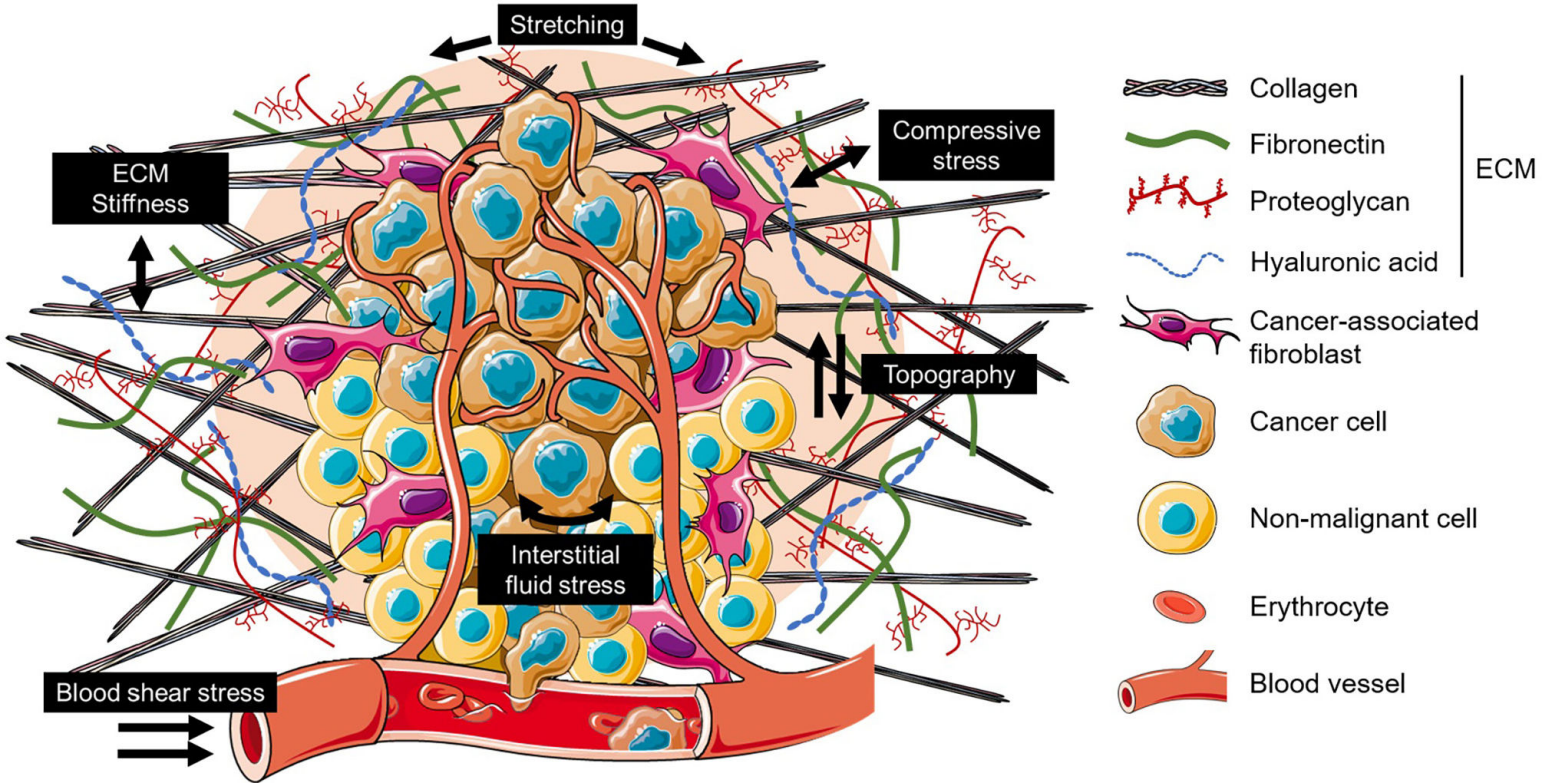
Example of mechanical forces applied on cells and tissue in tumor microenvironment. The extracellular matrix (ECM), the major acellular component of the TME, stiffens and reorganize itself under the impulsion of stromal cells, thus creating new mechanical stimuli. Moreover, the anarchic proliferation of cancer cells in a confined space also creates a compressive stress and a mechanical stretching on the TME. Finally, fluid shear stress is also present at the tumor level through the movement of interstitial fluid or in the capillaries and lymphatic vessels through blood and lymph circulation. Together, these forces drive the TME phenotype and have key impacts on differentiation, proliferation, migration, invasion and metastasis dissemination.

**Table 1:** Current studies having evaluated a direct effect of mechanical forces on the cellular radiation response.

Mechanical stress	Experimental system	Applied force	Radiation type (dose)	Time post IR	Cell lines	Significant outcomes	References

Stiffness	Fibronectin- coated polyacrylamide (PAA) hydrogel	Glass (>1 Gpa), 30, 20, 10,1 & 0.5 kPa	X-ray (1–10 Gy)	1–48 h	Human embryonic kidney (HEK293)Non-malignant breast (MCF10A)Breast adenocarinoma (MDA-MB-231)Breast cancer (MCF7) Adenocarcinomic alveolar basal epithelial (A549)Bone Osteosarcoma epithelial (U2OS)	– Stiffness < 1 kPa ↓ clonogenic survival and ↑ apoptosis after irradiation in all cell lines – Stiffness < 1 kPa ↑ γH2AX foci/cell at 24 h after 2 Gy in HEK293 cells – Stiffness < 1 kPa ↓ NHEJ and HR pathway efficiency in HEK293 cells – Stiffness < 1 kPa ↓ FK2, RNF168, 53BP1 and BRCA1 foci/cell at 1 h after 1 Gy in HEK293 cells – Stiffness < 1 kPa ↓ DSB repair efficiency and ↑ radiosensitivity through MAP4K4/6/7 kinase-mediated ubiquitin phosphorylation	Deng et al. [105]
Stiffness	Collagen-coated PAA hydrogel	0.5, 5 & 25 kPa	γ- Ray-^60^Co (2–8 Gy)	120 h	Cervical squamous cell carcinoma (SiHa)	– Low stiffness ↓ clonogenic survival after irradiation – 0.5 kPa stiffness ↑ apoptosis rate and cas-pase-3 expression level after 4 Gy	Jin et al. [104]
Stiffness	Collagen-coated PAA hydrogel	1.3 & 13 kPa	X-ray (2 & 10 Gy)	1(d1) & 3 (d3) days	Non-malignant breast (MCF10A)Breast adenocarinoma (MDA-MB-231)	– 13 kPa substrate ↑ MDA-MB-231 cell and nuclei area at d3 after 10 Gy – 13 kPa substrate ↓ MCF10A spreading at d1 after irradiation – 1.3 kPa substrate ↑ MDA-MB-231 migration after 2 Gy at d1 but not at d3 – 1.3 kPa substrate ↓ MDA-MB-231 migration velocity and ↑ directional persistence after 10 Gy	Panzetta et al. [120]
Shear stress	Parallel-plate flow chamber	Laminar flow (12 dyne/cm^2^)	X-ray (8 Gy)	3 & 24 h	Colon cancer (T84) Colon cancer (SW480)	– Shear stress for 24 h but not 3 h enhanced radiation-induced apoptosis and clonogenic cell death – Shear stress regulated radiosensivity via integrin p1/FAK signal pathway	Luo et al. [142]
Shear stress	Parallel-plate flow chamber	Laminar flow (16 dyne/cm^2^)	γ- Ray-^137^Cs (2 Gy)	24 h	Human aortic endothelial cells (HAECs)	– Shear stress ↑ radiation-induced endothelial nitric oxide synthase expression – Shear stress ↑ NF-kB signaling and ↓ oxidative stress signaling genes expression after irradiation	Natarajan et al. [144]
Stretching	PDMS membrane glued in a stretch chamber	10 % uniaxial cyclic stretch (0.5 Hz for 12 h)	UV-C at 45 J/m^2^	NS	Mouse 3 T3 fibroblasts	– Stretching ↓ γH2AX fluorescence intensity after UV irradiation	Nagayama et al. [158]
